# Comparative Study of Technologies for Tubule Occlusion and Treatment of Dentin Hypersensitivity

**DOI:** 10.3390/jfb12020027

**Published:** 2021-04-27

**Authors:** Camilla Berg, Erik Unosson, Håkan Engqvist, Wei Xia

**Affiliations:** Department of Materials Science and Engineering, Division of Applied Materials Science, Uppsala University, 751 21 Uppsala, Sweden; camilla.berg@angstrom.uu.se (C.B.); unosson.erik@gmail.com (E.U.); hakan.engqvist@angstrom.uu.se (H.E.)

**Keywords:** dentin hypersensitivity, occlusion, desensitizing agents, calcium phosphate, remineralization

## Abstract

This study aimed to evaluate the occluding/remineralization performance and resistance to acid attacks of the mineralization layer formed by a tooth-desensitizing gel containing amorphous calcium magnesium phosphate (ACMP) particles and compare it to six other desensitizing products available on the market. Similar comprehensive studies are few and there is especially a lack of studies that are up to date. A dentin-disc model was used for in vitro evaluation of the desensitizing toothpastes/gels. Application of the products was performed twice daily for seven days. One set of specimens were evaluated using scanning electron microscopy (SEM) directly after the final treatment and another set was evaluated after an acid challenge, exposing specimens to 2 wt% citric acid. The ACMP desensitizing gel was the only product resulting in complete occlusion by the formation of mineralized material on the dentin surface and inside the tubules. Particle deposition was dominant after treatment with the other desensitizing products, with little or no mineralization, resulting in partial occlusion only. Sensodyne Repair & Protect and Oral-B Pro-Expert showed the highest resistance toward acid attacks. Material inside the tubules remained relatively unaffected by acid attacks in all specimens. The results in this study indicated a great variability among the occluding agents in terms of occlusion and acid resistance of the mineralization layer. The high degree of occlusion and intra-tubular mineralization that could mitigate the effect of acid solubilization indicate that the ACMP desensitizing gel may be a superior option for the treatment of dentin hypersensitivity.

## 1. Introduction

Dentin hypersensitivity is characterized by a short and sudden pain caused by exposure of dentin as a result of loss of enamel or cementum [1]. The number of individuals who experience dentin hypersensitivity varies across studies, due to differences in study designs. Most studies, however, conclude that up to 57% of adult individuals and 84.5% of patients after periodontal treatment suffer from dentin hypersensitivity [2].

The underlying cause for dentin hypersensitivity has been widely discussed but the most used and commonly accepted explanation is the hydrodynamic theory [3]. It states that pain is elicited from exposed dentin due to fluid movement within the dentin tubules in response to stimuli, typically thermal, evaporative, tactile, osmotic, or chemical [4]. The movement of fluid results in mechanical deformation of the nerve terminals in the pulp, which causes pain [5].

One way of treating dentin hypersensitivity is to hinder, or reduce, the movement of fluids within the tubules. This can be achieved in several different ways ranging from invasive techniques such as laser etching of the dentin surface to non-invasive techniques that can be applications of a gel or toothpaste containing an occluding agent [6,7]. Occluding agents can be particles that either physically block the tubules and/or particles or ions that can induce the formation of a new mineralized layer.

An efficient occluding agent should offer fast pain relief and be resistant to acid attacks to be able to offer a long-lasting effect. Fast pain relief can be achieved if a large number of particles is deposited on the dentin surface or if the formation of the mineralized layer is rapid. Acid attacks occur upon exposure to low pH, leading to acid erosion of enamel and dentin, and can be compared to drinking an acidic beverage, such as coffee or a fruit drink [8]. Acid resistance can be achieved if the occluding particles or the mineralized material can withstand dissolution when the pH decreases. Intra-tubular occlusion, attained by a small particle size or intra-tubular mineralization, can also help to reduce the effect of acid attacks [9,10].

In two previous studies, sub-micron spherical and hollow particles of amorphous calcium magnesium phosphate (ACMP) were shown to offer fast mineralization when applied via a desensitizing gel. A mode-of-action study disclosed that this was accomplished through the penetration of particles deep into the tubules and a fast and continuous release of calcium and phosphate ions that induced mineralization both on the dentin surface as well as inside the tubules [11,12].

Despite the number of available treatment options for dentin hypersensitivity, there is no comprehensive, up-to-date, in vitro comparison of the occluding performance of products intended for at-home use. Differences in study design make it difficult to compare the efficiency between products. The aim of the current study was, therefore, to qualitatively compare the effect of the ACMP particles with six other technologies that are commercially available for the treatment of dentin hypersensitivity. This was done with respect to dentin tubule occluding performance and resistance toward acid attacks when using an in vitro dentin-disc model.

## 2. Materials and Methods

### 2.1. Desensitizing Products

Six currently available commercial products with dentin tubule occluding/remineralization claims were included in the study to compare the performance of the ACMP desensitizing gel. Technology and mode of action for each product are listed in Table 1. Each product was used on two separate dentin specimens, one for direct evaluation and one for evaluation after treatment followed by an acid challenge.

### 2.2. Sample Preparation

Extracted human molars, free from caries and without anatomical defects, were used in the study to compare the occlusion effects and resistance to acid attacks. The use of human molars was performed in accordance with the guidelines from the Swedish Ethical Review Authority (2016/039). Specimens were prepared by cutting 1-mm-thin discs from the midsections of the coronal part of the molars using a low-speed saw (Buhler Isomet 2000). The tubules were exposed and the smear layer removed by etching the specimens in 35% phosphoric acid for 15 s, followed by thorough rinsing in deionized water.

### 2.3. Treatment Sequence

All specimens were treated twice daily for a total of seven days. Specimens were gently dried using a low-lint tissue, and approximately 0.6 g of the desensitizing products were applied to the dentin surface with a soft-bristled toothbrush (GUM SensiVital), using a circular motion and light hand pressure. Both sides of the specimens were brushed for 30–45 s each, and excess material was removed using the toothbrush. The specimens treated with MI Paste Plus were treated according to instructions from the manufacturer, i.e., application of the product using a gloved finger. The paste was applied evenly on both sides and left undisturbed for 3 min after which excess gel was removed. All products, apart from the ACMP gel, contained fluoride (900–1450 ppm F), for which treatment with a standard fluoride toothpaste (Pepsodent Super Fluor, “PSF”, 1450 ppm F) was performed prior to each application of the ACMP gel. This was performed by applying approximately 0.6 g of PSF on the dentin specimen and brushing both sides of the specimens as previously described. All specimens were stored in individual containers in 2 mL of complete artificial saliva (T0300, Northeast Laboratory Services, Winslow, Maine, USA) at 37 °C on a rocking platform in between treatments. The artificial saliva was exchanged daily, and the samples were vacuum dried at the end of the period of treatment (after seven days).

### 2.4. Acid Challenge

After the final application and storage in saliva at 37 °C overnight, one set of specimens was subjected to an acid challenge by immersing the specimens in 2 wt% citric acid (pH 2) for 30 s to mimic the exposure to an acidic beverage. The specimens were then rinsed thoroughly in deionized water for 2 min and gently dried using a low-lint tissue followed by vacuum drying.

### 2.5. Characterization

The specimens were characterized with scanning electron microscopy (SEM; Zeiss, Leo 1530), imaging the samples with secondary electrons at 2 kV acceleration voltage. To avoid charging and to allow for imaging, the specimens were sputtered with a conductive Au/Pd layer prior to analysis. Cross sections of the dentin specimens were analyzed by manually fracturing the dentin discs longitudinally.

## 3. Results

### 3.1. Occlusion

**Figure 1:**
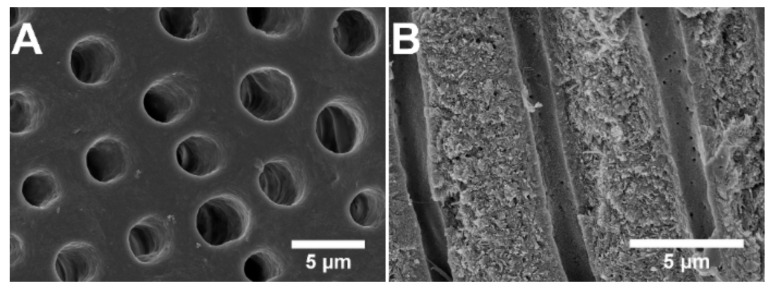
SEM micrographs of an untreated dentin specimen showing (**A**) the surface and (**B**) a cross section of the same specimen ~30 µm from the surface.

Figure 1 shows the typical appearance of an etched dentin specimen with exposed tubules, both on the surface of the dentin disc and when examining the cross section. The SEM micrographs show that the tubules were open both on the surface and along the depth of the tubules.

**Figure 2:**
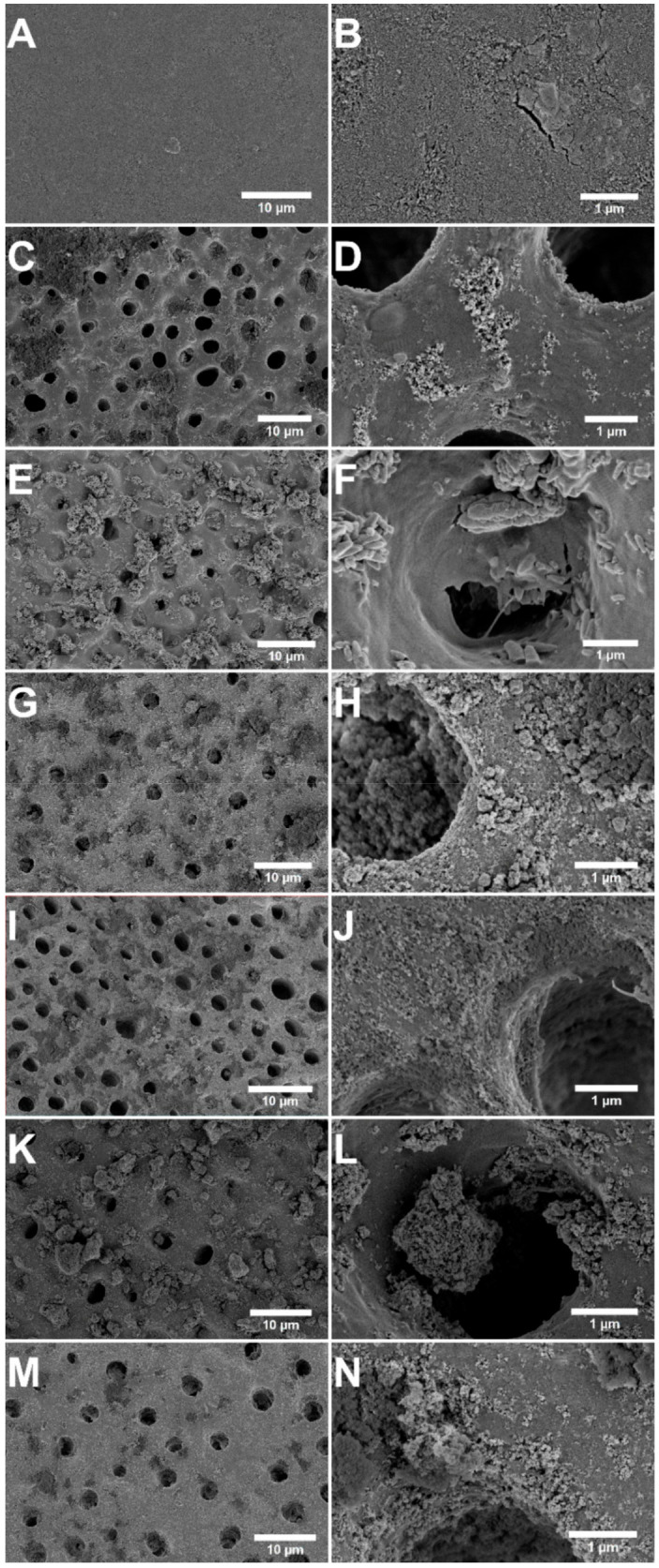
SEM micrographs of the dentin surface of the samples treated with (**A**,**B**) PSF + ACMP gel, (**C**,**D**) Sensodyne Repair & Protect, (**E**,**F**) Colgate PRO-Relief, (**G**,**H**) Oral-B Pro-Expert, (**I**,**J**) MI Paste Plus, (**K**,**L**) GUM SensiVital+, and (**M**,**N**) Enamelon Gel. Left panels show images at low magnification and right panels show images taken at higher magnification.

**Figure 3:**
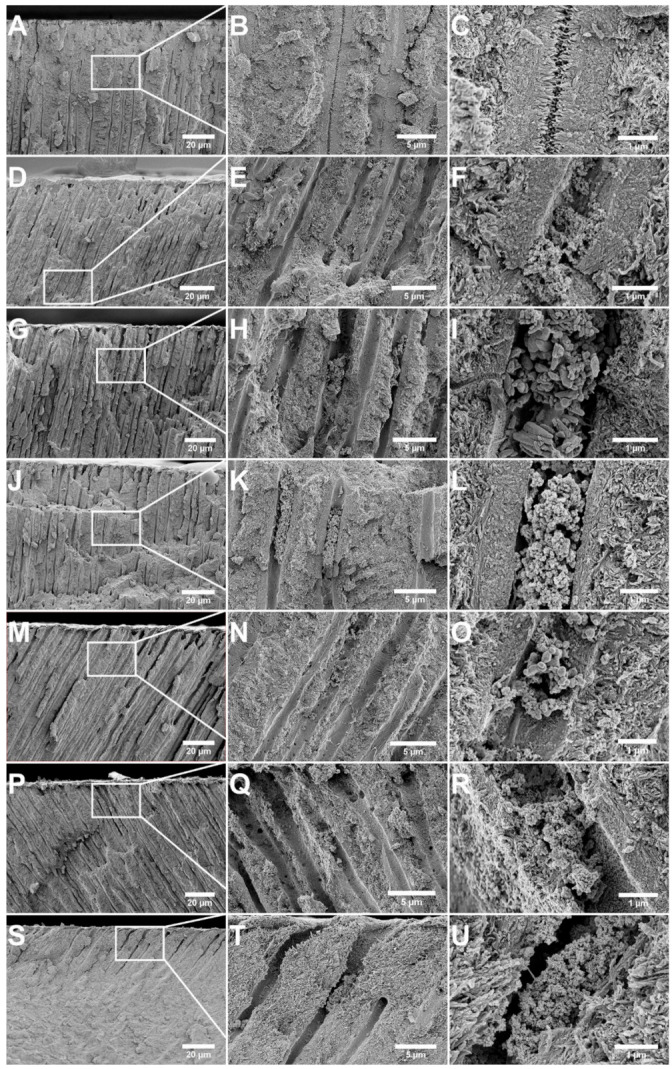
SEM micrographs of the cross sections of the samples treated with (**A**–**C**) PSF + ACMP gel, (**D**–**F**) Sensodyne Repair & Protect, (**G**–**I**) Colgate PRO-Relief, (**J**–**L**) Oral-B Pro-Expert, (**M**–**O**) MI Paste Plus, (**P**–**R**) GUM SensiVital+, and (**S**–**U**) Enamelon Gel. The two left panels show images at low magnification and mid, respectively, and the right panels show images taken at higher magnification.

The results from the in vitro tubule occlusion for the seven occluding agents evaluated in this study are summarized in Table 2. The specimen treated with PSF and the ACMP gel exhibited complete occlusion, and the surface was entirely covered by a newly formed mineralized layer (Figure 2A,B). Cracks appeared on the mineralized surface of the occluded tubules, which were most likely a result of drying the specimen under vacuum. The cross section of the same specimen demonstrated that the tubules were completely occluded to a depth of approximately 30 µm from the dentin surface (Figure 3A). High-magnification images of the tubule cross section showed that the crystals residing within the tubules were needle-like, primarily oriented toward the center of the tubule. No distinct interface between the mineralized material and the tubule walls was observed, indicating that the mineralized material was highly integrated and extending out from the tubule walls (Figure 3B,C).

Observation of the surface of the dentin after treatment with Sensodyne Repair & Protect showed deposition of Bioglass particles and aggregates larger than the tubule openings (Figure 2C,D). This caused the occlusion to be limited to the dentin surface, with the majority of the tubules still exposed even after the treatment. Some material, in the form of clusters of small, rounded particles (~10–20 nm in diameter), was located inside the tubules, forming a plug, but there was no apparent integration with the peritubular dentin (PTD, Figure 3D–F) that makes up the tubule wall. No significant mineralization was observed either on the surface of the sample or inside the tubules.

Images in Figure 2E,F demonstrate the appearance of the dentin surface after treatment with Colgate PRO-Relief toothpaste. The tubules were partly occluded and many were filled/plugged with deposited, irregularly shaped particles (200 nm–1 µm long, Figure 2F) but without any evident mineralization on the surface. The cross section revealed that the tubules were still open and there was no integration between the particles that had penetrated the tubules and the tubule walls (Figure 3G–I).

After treatment with Oral-B Pro-Expert toothpaste, the dentin surface was covered with plenty of material and some tubules were occluded with stannous fluoride particle matrix (clusters of small spherical particles with a diameter of ~10–20 nm) (Figure 2G,H). Some particles were found to be located deep inside the tubules when examining the cross section of the sample, forming a plug that blocked the tubule, but there was no integration with the tubule wall. Many of the tubules remained exposed after the treatment (Figure 3J–L).

Treatment with MI Paste Plus topical cream resulted in deposition of some material on the dentin surface and inside the tubules, but the occlusion was generally poor (Figure 2I,J). There was no substantial integration between the CPP-ACP particles (100–200 nm in diameter) found in the tubules and the PTD, which can be seen in Figure 3M–O.

Figure 2K,L and Figure 3P–R demonstrate the effect of treatment with the Sunstar GUM SensiVital+ toothpaste containing HA particles. A large number of particles was deposited on the surface, essentially blocking a share of the tubules. Some mineralization on the surface occurred, but most of the tubules remained exposed. The cross section of the specimen revealed a thin (~2 µm) occlusion layer on the dentin surface as well as some particles (50–100 nm) lodged inside the tubules (Figure 3P–R). Most of the tubules were, however, still open and there was only a minor integration between the particles found inside the tubules and the tubule wall.

The specimen treated with the Premier Enamelon Preventive Treatment gel exhibited some surface mineralization and partial occlusion, but the tubules were still largely exposed (Figure 2M,N). Deposits of nano-scaled particles and aggregates of the same were found adhering to the dentin surface. Cross-section images, seen in Figure 3S–U, demonstrate the presence of a plug, but a majority of the tubules were still open. Partial mineralization of the tubule walls was noted, but integration between the particle deposits and surrounding tissue was generally poor.

### 3.2. Acid Challenge

**Figure 4:**
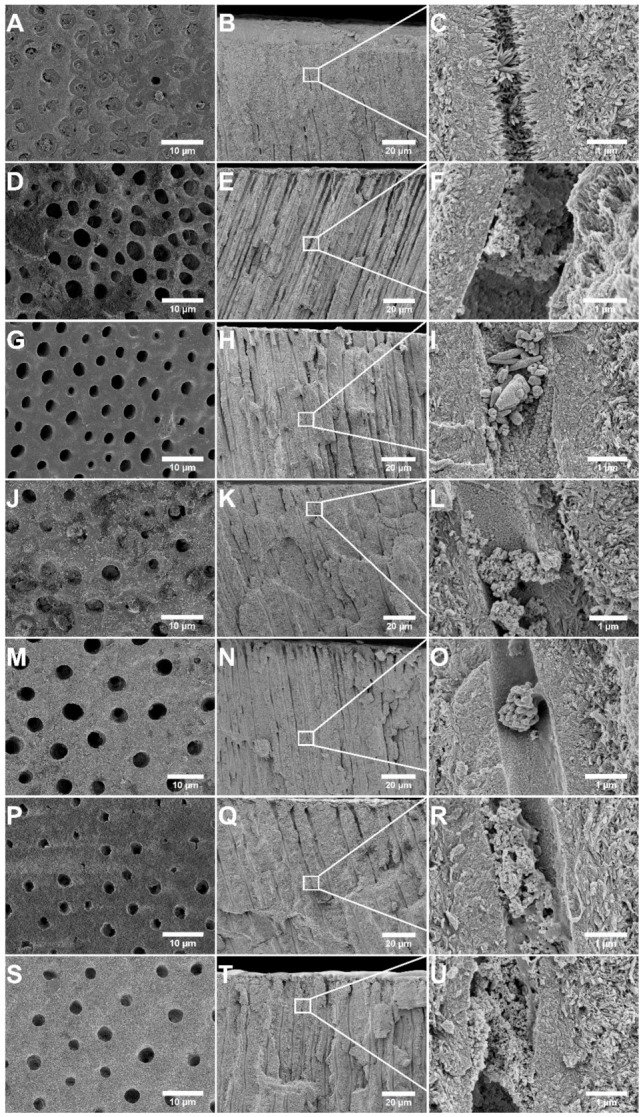
SEM micrographs of the dentin surface after acid challenge with 2 wt% citric acid for 30 s. (**A**–**C**) PSF and ACMP gel, (**D**–**F**) Sensodyne Repair & Protect, (**G**–**I**) Colgate PRO-Relief, (**J**–**L**) Oral-B Pro-Expert, (**M**–**O**) MI Paste Plus, (**P**–**R**) GUM SensiVital+, and (**S**–**U**) Enamelon Gel. Left panels show images at low magnification and mid, and the right panels show the cross section of the same specimen at low and higher magnification.

The appearance of the dentin specimens after twice-daily treatment for seven days, followed by an acid challenge, is shown in Figure 4. It is apparent from the surface evaluation of the sample treated with PSF and ACMP gel that the acid etched through the mineralized layer, since many of the tubule openings reappeared (Figure 4A). Although visible, essentially all tubules were still occluded with mineralized material across and inside the tubules. The cross-section evaluation verified that the tubules were still occluded below the dentin surface, and no effect could be seen on the intra-tubular mineralized material (Figure 4B,C).

Figure 4D–F shows the appearance after the acid challenge of the sample treated with Sensodyne Repair & Protect toothpaste. Compared to the surface before the acid challenge (Figure 2C,D), a larger share of the tubules was exposed, indicating that the acid had dissolved some of the mineralized material on the surface. Particles presumed to be Bioglass particles were still present on the surface.

The dentin specimen treated with Colgate PRO-Relief toothpaste was heavily affected by the acid challenge, as shown in Figure 4G–I. Essentially all tubules were re-exposed, and none of the deposited particles seen in Figure 2E,F remained on the surface. The particles inside the tubules were less affected by the acid exposure, but the intra-tubular occlusion was still poor since the amount of particles lodged inside the tubules after the treatment was small (Figure 3G–I).

The Oral-B Pro-Expert specimen fared comparably well in the acid challenge, with no significant changes in terms of tubule occlusion or particle deposits on the surface (see Figure 4J–L compared to Figure 3G,H). The sample treated with GC MI Paste Plus did, in contrast to this, withstand the acid challenge poorly. The surface-adhered particles had dissolved and the tubules were again exposed (Figure 4M–O).

The specimen treated with GUM SensiVital+ that was subjected to acid challenge is shown in Figure 4P–R. Compared to the images shown in Figure 3K,L, there were no particles on the surface, but a layer of mineralized material was still present. However, this mineralized layer did not occlude the tubules effectively.

The dentin surface and cross-section appearance after treatment with Enamelon Gel and the acid challenge are shown in Figure 4S–U. Some nano-scaled particles still adhered to the surface, but all larger particle aggregates had dissolved or vanished, and the tubules were largely exposed.

## 4. Discussion

The hydrodynamic theory is widely accepted as the principal mechanism of action for the cause of dentin hypersensitivity [3]. Occlusion of exposed dentin tubules can reduce pain related to the condition by the hindrance of fluid movements within the dentin tubules [5]. In this study, the occluding effect and resistance to acid attacks were evaluated for seven different desensitizing products after a twice-daily, seven-day treatment period.

Examining the results, in general, it was only the specimen treated with PSF and ACMP gel that resulted in complete occlusion with a mineralized layer covering the entire dentin surface (Figure 2A,B). In our previous studies, we showed that the ACMP particles alone result in this type of occlusion. Fluoride application does not affect the degree of occlusion upon application, but it alters the mineralization characteristics by the formation of needle-like structures [11,12]. The sample treated with PSF and ACMP gel was additionally the only sample that exhibited intra-tubular mineralization with good adherence of the occluding material and the tubule walls (Figure 3A–C). As described by Markowitz and Pashley, the key factor in reducing the hydraulic conductance, achieving pain-relief, is to reduce the anatomic tubule radius [22]. This indicates that the occlusion by mineralization after treatment with PSF and ACMP gel would offer a higher degree of pain relief compared to the samples with less intra-tubular occlusion or poor integration between the occluding particles and the tubule wall. As reported by Ryou et al. [23], there is a natural variation in tubule diameters related to age and the gradual formation of sclerotic dentin, which reduces the tubular lumen diameter. The current study was based on a limited set of extracted molars obtained from separate individuals. However, the tubule diameters were generally in agreement across specimens when examining unaffected areas further from the treated surface in cross section (Figure 3 and Figure 4). This supports the claim that the mineralization and reduction of the anatomic tubule diameter was, in fact, a result of the treatment with PSF and the ACMP gel.

The other treatment options that resulted in mineralization, at least on the dentin surface, were GUM SensiVital+ and Premier Enamelon containing HA particles or ACP (Figure 2K–N). The probable reason for this is that they, in a similar manner as the ACMP particles, release calcium and phosphate ions to a concentration exceeding the supersaturation of saliva, triggering the nucleation of material on the dentin surface [19,20,21]. Previous studies using NovaMin^®^ Bioglass and CPP-ACP reported that the mineralization was caused by the release of previously mentioned ions, which were not clearly observed in this study [24,25,26,27]. Only particle deposition was observed in all three cases, with no significant surface or intra-tubular mineralization. This could possibly be explained by inter-study variations in the design of the studies, such as the amount of gel applied on each brushing, storage conditions, or the number of times that the gel has been applied.

No mineralization was observed for the specimens treated with Colgate PRO-Relief (Figure 2E,F and Figure 3G–I) or Oral-B Pro-Expert (Figure 3G,H and Figure 4J–L), which can be explained by the lack of phosphate ions in the toothpastes. Arginine is added to the Colgate PRO-Relief toothpaste since the association of calcium carbonate, and the amino acid is said to provide an alkaline environment that can encourage endogenous calcium and phosphate ions to deposit on the dentin surface [28]. The results in this study, however, indicated that this effect, if present at all, was much slower compared to direct delivery of calcium and phosphate ions. The stannous fluoride particles in Oral-B Pro-Expert are said to offer pain relief by particle deposition on the dentin surface, so the lack of a mineralized layer was expected for this specimen [16].

The resistance toward acid attacks varied greatly when comparing the different treatment alternatives (Figure 4). This is most likely dependent on the solubility of the occluding material, particularly at low pH. The occluding materials that were the least affected by the acid challenge were the NovaMin^®^ Bioglass particles in Sensodyne Repair & Protect and the stannous fluoride particles in the Oral-B Pro-Expert toothpaste (Figure 4D–F,J–L). These results were in accordance with previous studies using Bioglass and stannous fluoride complexes (with hexametaphosphate) as occluding agents, where both materials were shown to resist acid solubilization [13,29,30]. The HA particles included in the GUM SensiVital+ toothpaste also had fairly low solubility, at least compared to other calcium phosphate phases, which was indicated, in part, by the mineralized layer that remained after acid challenge (Figure 4P–R) [31].

Specimens treated with toothpastes containing, or claiming to form, ACP (i.e., MI Paste Plus and Premier Enamelon) had a high solubility and are, thus, susceptible to acid attacks [31]. This was clearly visible in Figure 4M–O,S–U. The Colgate Pro-Argin particles (arginine with calcium carbonate) appeared to be particularly sensitive to acid since all particles that largely covered the dentin surface before the acid treatment (Figure 2E,F) were removed by the exposure to acid (Figure 4G–I). This can be explained by the solubility of calcium carbonate that is higher compared to calcium phosphate, which could withstand acid attacks better. The solubility of calcium carbonate is pH-dependent due to the fact that it reacts with the acid to form calcium ions, water, and carbon dioxide, even in dilute acidic solutions [32]. Apatite materials, either in particle form or as a mineralized layer, were, in comparison, not as sensitive to acid exposure [33,34].

The particles in the ACMP gel are, like the other ACP materials, highly soluble in aqueous solutions, particularly at low pH. This was confirmed already prior to the acid challenge where all particles had dissolved (Figure 2A,B). The high dissolution rate and consequent high release of ions, combined with deep penetration inside the tubules, make for a rapid crystallization process that occludes the tubules. Particle dissolution and release of phosphate ions will also raise pH, aiding the formation and stability of HA [31]. The acid resistance of the ACMP gel treated specimen should, therefore, not be assigned to the resistance of the particles themselves but to the crystalline and firmly integrated mineral that is rapidly formed inside the tubules by the transformation of the particles. The formation of needle-like structures (Figure 3C and Figure 4C) indicated that the acid resistance may have been further enhanced by the use of a fluoride toothpaste prior to the application of the ACMP particles. We showed in a previous study that fluoride incorporation in the mineralized material could be recognized by the formation of needle-like structures, similar to what was observed in this study [12]. This would improve the resistance toward acid attacks since fluoride substitution in apatites is known to increase the stability through the reduction of strain in the crystal lattice [31,35].

It should be noted that this study only was performed in terms of evaluating the qualitative differences of the occluding agents, their effects on remineralization of dentin, and resistance to acid attacks after a single week’s daily application. For quantitative comparisons, more tests have to be performed. Reeder et al. developed a setup for measuring the hydraulic conductance, i.e., the ease with which a fluid can “filter” across dentin, which can be used to determine the occlusion in a quantitative manner [36]. This technique has successfully been used in several other comparative studies of occluding agents [37,38,39].

## 5. Conclusions

Given the timeframe and design of the current in vitro study, particle deposition was dominant for all evaluated toothpastes/gels except for the ACMP desensitizing gel that induced the significantly better formation of a mineralized layer occluding exposed dentin tubules. The resistance toward acid attacks was highest for Sensodyne Repair & Protect and Oral-B Pro-Expert, but the risk of re-exposure of the dentin tubules could likely be mitigated by intra-tubular mineralization, as observed after application of the ACMP gel. Suggesting that the key factor for an efficient treatment is the formation of a mineralized layer, the results in this study indicate that the gel containing the ACMP particles may be a promising alternative for the treatment of dentin hypersensitivity. This warrants further investigations of the occluding agent, i.e., quantitative comparisons with competing products and clinical evaluation.

## Figures and Tables

**Table 1:** Sample matrix showing the products (toothpastes/gels) included in the study, the technologies, and the mode of action assigned to each product.

Product	Technology	Mode of Action
PSF * + ACMP gel	Magnesium stabilized amorphous calcium phosphate—ACMP (CAPOSAL^®^)	ACMP particles induce mineralization on the dentin surface and tubules by deep penetration of the particles and rapid release of calcium, magnesium, and phosphate ions that elevate pH and crystallize into hydroxyapatite [11].
Sensodyne Repair & Protect (GlaxoSmithKline)	Bioglass (NovaMin^®^)	Bioglass particles interact with saliva, which results in an increase in pH, release of bioavailable calcium and phosphate ions, resulting in a mineralized layer on the dentin surface [13].
Colgate Sensitive PRO-Relief (Colgate-Palmolive)	Arginine (Pro-Argin^®^)	Arginine and calcium carbonate form a positively charged complex that binds to the negatively charged dentin surface, which physically blocks the tubule openings [14,15].
Oral-B Pro-Expert (Procter&Gamble)	Stannous fluoride	Deposition of stannous fluoride complexes on the dentin surface that occlude exposed tubules [16].
MI Paste Plus (GC Corporation)	Casein phosphopeptide amorphous calcium phosphate—CPP-ACP (RECALDENT™)	CPP-ACP adheres to dentin and releases bioavailable calcium and phosphate ions to aid remineralization [17,18].
GUM SensiVital+ (Sunstar)	Hydroxyapatite—HA	HA particles occlude exposed tubules and release calcium and phosphate ions for remineralization of the dentin surface [19].
Enamelon Preventive Treatment Gel (Premier)	Amorphous calcium phosphate—ACP	Stannous fluoride together with calcium and phosphate salts form ACP in situ that occludes and mineralizes exposed tubules [20,21].

* Pepsodent Super Fluor toothpaste.

**Table 2:** Summary of the results from the treatment using the different desensitizing products.

Product	Occlusion	Material on the Surface	Material inside the Tubules	Mineralization	PTD * and Material Integration
PSF ** + ACMP gel	Complete	Dense mineralized	Needle-like	Yes (surface + tubules)	Yes
Sensodyne Repair & Protect	Partial	Bioglass particles (5–10 µm) + aggregates of small rounded particles (10–20 nm)	Small rounded (10–20 nm)	No	No
Colgate Sensitive PRO-Relief	Partial	Irregularly shaped particles (200 nm–1 µm) + aggregates	Irregularly shaped particles (200 nm–1 µm)	No	No
Oral-B Pro-Expert	Partial	Small rounded particles (10–20 nm) + aggregates (500 nm–10 µm)	Small rounded particles (10–20 nm)	No	No
MI Paste Plus	Poor	Round particles (100–200 nm) + small rounded particles (10–20 nm)	Round (100–200 nm)	No	No
GUM SensiVital+	Partial	Small irregularly shaped particles (50–100 nm) + aggregates (1–3 µm)	Small irregularly shaped (50–100 nm)	Yes (surface)	No
Enamelon Preventive	Partial	Small rounded particles (10–20 nm) + aggregates (1–2 µm)	Small rounded (10–20 nm)	Yes (surface)	Poor

* Peritubular dentin, ** Pepsodent Super Fluor toothpaste.

## Data Availability

The data presented in this study are available on request from the corresponding author.
